# Functions of Redox Signaling in Pollen Development and Stress Response

**DOI:** 10.3390/antiox11020287

**Published:** 2022-01-30

**Authors:** Dong-Ling Xie, Xue-Lian Zheng, Can-Yu Zhou, Mukesh Kumar Kanwar, Jie Zhou

**Affiliations:** 1 Department of Horticulture, Zhejiang University, Yuhangtang Road 866, Hangzhou 310058, China; 12116061@zju.edu.cn (D.-L.X.); 12016057@zju.edu.cn (X.-L.Z.); 22116057@zju.edu.cn (C.-Y.Z.); kanwar@zju.edu.cn (M.K.K.); 2 Key Laboratory of Horticultural Plants Growth, Development and Quality Improvement, Agricultural Ministry of China, Yuhangtang Road 866, Hangzhou 310058, China; 3 Shandong (Linyi) Institute of Modern Agriculture, Zhejiang University, Linyi 276000, China

**Keywords:** environmental stress, oxidants, pollen, redox signaling, ROS

## Abstract

Cellular redox homeostasis is crucial for normal plant growth and development. Each developmental stage of plants has a specific redox mode and is maintained by various environmental cues, oxidants, and antioxidants. Reactive oxygen species (ROS) and reactive nitrogen species are the chief oxidants in plant cells and participate in cell signal transduction and redox balance. The production and removal of oxidants are in a dynamic balance, which is necessary for plant growth. Especially during reproductive development, pollen development depends on ROS-mediated tapetal programmed cell death to provide nutrients and other essential substances. The deviation of the redox state in any period will lead to microspore abortion and pollen sterility. Meanwhile, pollens are highly sensitive to environmental stress, in particular to cell oxidative burst due to its peculiar structure and function. In this regard, plants have evolved a series of complex mechanisms to deal with redox imbalance and oxidative stress damage. This review summarizes the functions of the main redox components in different stages of pollen development, and highlights various redox protection mechanisms of pollen in response to environmental stimuli. In continuation, we also discuss the potential applications of plant growth regulators and antioxidants for improving pollen vigor and fertility in sustaining better agriculture practices.

## 1. Introduction

As sessile organisms, plants are more susceptible to environmental influences than animals. In order to survive and reproduce, they need to constantly adjust their physiology and morphology to adapt to various environmental challenges, which often cause cellular oxidative damage. Therefore, plants have evolved complex oxidative stress regulation systems, such as Heat Shock Protein (HSP) networks, endoplasmic reticulum-unfolded protein response (ER-UPR), cytosolic protein response (CPR), Ca^2+^ signaling, and redox regulation systems [1,2]. Among them, cellular redox regulation plays an indispensable role. Redox biology results from the modification of target proteins by reactive oxygen species (ROS) and other oxidants, which, in turn, affect protein structure, function, and signal transduction [3]. Plants possess a large network of ROS producing/scavenging pathways involving more than 100 genes to strictly maintain the appropriate cellular redox status [4]. Various antioxidants in plant cells keep oxidants at non-toxic levels, and any change in this balance could serve as a redox signal [5]. As long as the concentration of ROS in plant cells remains within the normal range, the normal cellular metabolic activities can carry on.

**Figure 1:**
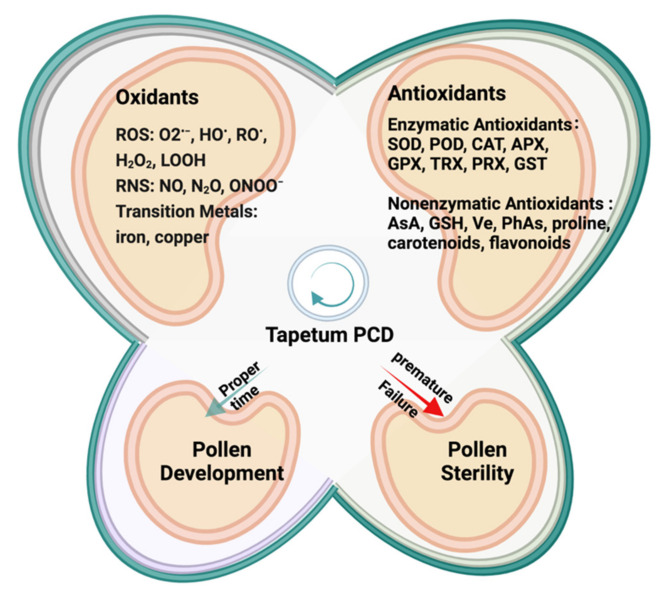
Redox biomolecules involved in pollen development. The normal development of pollen requires timely degradation of the tapetum, and this process is very susceptible to the disturbance of redox homeostasis. Premature or failure of tapetum degradation will lead to microspore abortion and pollen sterility. In the development and regulation of pollen and anthers, the main oxidants contain reactive oxygen species (ROS) and reactive nitrogen species (RNS), and the antioxidants can be divided into enzymes and non-enzymatic antioxidants.

Pollen plays a key role in the life cycle of flowering plants and is directly related to plant fertility and crop productivity. However, pollen development is very susceptible to environmental influences, so plants have evolved intricate mechanisms to ensure the smooth completion of this process. Studies on reproductive development have uncovered a clear link between redox processes and pollen development [6,7]. Pollen development is completed in anthers and is generally divided into three stages: microsporogenesis, male gametophyte formation, and pollen maturation [8]. The coordinated development of anthers and pollen is necessary for the successful reproduction of plants. Tapetum is a layer of cells in anther, which maintains high metabolic activity during microsporogenesis. Tapetum cells secrete proteins, lipids, carbohydrates, and secondary metabolites through programmed cell death (PCD) to provide nutrients and other essential substances for pollen development [9,10,11]. The distortion of tapetum morphology and the change in tapetum degradation time usually cause pollen deficiency, which further affects the number and morphology of pollen, the structure of pollen wall and pollen fertility [1]. Numerous studies have shown that tapetal PCD is induced by the ROS homeostasis. Transient peaks of ROS production have been observed in the developing anthers of rice (*Oryza sativa*), tomato (*Solanum lycopersicum*), and tobacco (*Nicotiana tabacum*) [12,13]. However, excessive accumulation of ROS indiscriminately damages cellular constituents, including proteins, DNAs, and lipids, and leads to atypical tapetal PCD and defective pollen wall formation [3]. As we have seen, redox regulation and signaling have come into sight as crucial mechanisms that regulate the sexual reproduction of plants, in which ROS, nitric oxide (NO), and other classical antioxidants and biomolecules are closely involved [14]. These redox components (Figure 1) are essential for maintaining redox homeostasis and ensuring normal anther development and pollen germination [15,16].

Abiotic stresses such as heat, cold, and drought can induce oxidative stress in plant cells, resulting in the imbalance of cellular redox system. In most plants, meiosis to microspore development seems to be the most sensitive process to environmental stress conditions [17]. The stresses during this period often lead to microspore abortion, reduced number of pollen grains during flowering, and a reduction in the proportion of fertile pollen [18]. In the process of evolution, pollen has developed a different and complicated mechanism in response to environmental oxidative stresses. On the one hand, it shares the similar redox regulation mechanisms with other vegetative and reproductive processes. Excessive accumulation of oxidants such as ROS can cause irreversible cell damage, but can also act as signals to trigger a variety of stress responses [19,20], including increasing the expression of antioxidant genes and the production of enzymatic/non-enzymatic antioxidants, strengthening the repair and recovery of denatured proteins, and ultimately enhancing tolerance in plants [1,17]. On the other hand, pollen has its specific redox mechanisms. Specific alterations in phospholipid saturation and accumulation of chaperonin HSPs, sugars, and nonprotein amino acids (such as proline) are identified to regulate pollen oxidative stresses [1,21].

## 2. Redox Biology in Pollen Development

### 2.1. Role of Oxidants in Pollen Development

The major endogenous sources of oxidants in plants (Figure 1) are various intermediates of oxygen, nitrogen, and carbon metabolism including ROS, reactive nitrogen species (RNS), reactive carbonyl species (RCS), and transition metals such as iron and copper [22]. ROS and RNS are the most common oxidants and are key regulators of plant development [7].

ROS are a collective term that includes superoxide (O_2_**^•^**^−^), the hydroxyl radical (HO^•^), lipid alkoxy (RO**^•^**) radicals, hydrogen peroxide (H_2_O_2_), and lipid peroxides (LOOH) [23]. ROS levels are dynamic in anther development. They begin to accumulate in the early stage of meiosis, gradually increase in the meiotic stage, and reach their highest level in the late stage of microspores. However, their signals decrease from the mitotic stage to the anther dehiscence in different plant species, such as Arabidopsis (*Arabidopsis thaliana*), tomato, tobacco, and wheat (*Triticum aestivum*) [13,24,25].

The production of ROS is precisely regulated during pollen development. ROS, which are produced by NADPH oxidases, in plants known as Respiratory Burst Oxidase Homologs (RBOHs), are essential for PCD during tapetum degradation in Arabidopsis, rice, tomato, and tobacco [12,13,24,26]. Failure or premature PCD of tapetum frequently leads to male sterility [27,28]. Therefore, a complex transcriptional network has been found to be responsible for fine-tuning the expression of RBOHs, generating ROS signals for the tapetal PCD to ensure pollen development [24,29]. The genome of Arabidopsis encodes a minimum of 10 RBOHs, and they have distinct expression profiles in the different organs and at various developmental stages [30]. For example, *RBOHE* overexpression leads to precocious tapetum degeneration, while *RBOHE* deficiency shows delayed tapetum degradation, both of which lead to pollen fertility. The ROS levels in *rbohe* mutants are generally reduced, while the increase in ROS levels in *RBOHE* overexpression plants only exists in the early developmental stage of pollen, indicating the importance of temporal and spatial regulation of ROS levels for pollen development [24]. Moreover, *RBOHC* plays a redundant role in tapetal degeneration and pollen development compared with *RBOHE* [24]. In tomato, Brassinazole Resistant 1 (BZR1) regulates *RBOH1* transcription and *RBOH1*-mediated ROS directly trigger PCD and the degradation of tapetum cells, which, in turn, plays an important role in pollen and seed development [31].

In addition to the direct influence of RBOH proteins, many regulatory genes have been identified to be responsible for anther cell differentiation and pollen formation in Arabidopsis [8]. *Anther Dehiscence Repressor* (*ADR*), which is involved in regulating male sterility in Arabidopsis, is *N*-myristoylated and targeted to the peroxisome during the early stages of flower development to negatively regulate anther dehiscence by suppressing ROS accumulation [32]. Tomato *Inflorescence Deficient in Abscission* (*SlIDA*) encodes a 14-mer EPIP peptide, which plays an essential role in pollen development and pollen tube elongation by regulating ROS homeostasis. *Slida* mutants assume decreased fertility, PCD defects in tapetum and septum, and failure of anther dehiscence. Exogenous use of synthetic 14-mer EPIP peptide rescues this defect and enhances ROS-signaling [33]. Rice *ARGONAUTE 2* (*OsAGO2*) is highly expressed in anthers and regulates anther development by regulating the methylation levels of the promoter region of *Hexokinase 1* (*OsHXK1*), which controls the appropriate production of ROS and the proper timing of tapetal PCD [34].

The fine-tuning of ROS involves many other molecules and antioxidants. For instance, Metallothioneins (MTs) are Cys-rich proteins that can scavenge HO**^•^** and singlet oxygen. OsMT2b, a ROS scavenger during rice male reproduction, interacts with Defective tapetum cell death 1 (DTC1) and its activity is inhibited by DTC1, which can regulate tapetum PCD process and the normal development of pollen in rice [35]. OsMADS3 can directly up-regulate the expression of *MT-1-4b* to modulate ROS levels in rice male reproductive development [12].

In addition, fatty acids (FAs) are also involved in the process of pollen development and are closely related to the redox regulation [36,37,38]. In maize (*Zea mays*), lipid biosynthesis mediated by male sterility 33 (ZmMS33) is critical to the normal structure and function of chloroplasts in tapetum cells and the late proliferation and expansion of anther cells. The loss of function of ZmMS33 leads to the accumulation of H_2_O_2_ in the anthers, early and excessive autophagy, premature PCD of tapetum cells, and it affects the late development of anthers [28]. In Arabidopsis, the loss of *Fatty Acid Export* 1(*FAX1*) function breaks FA/lipid homeostasis and ROS homeostasis in the cells, suppresses transcriptional activation of anther developmental genetic networks, and damages tapetum development and pollen wall formation, leading to male sterility [38]. Consequently, the production of ROS is regulated at different levels at different stages, so as to maintain the balance of redox and the normal development of pollen.

RNS includes NO, nitrous oxide (N_2_O), peroxynitrite (ONOO^−^), and other types of nitrogen-containing reactive free radicals [22]. Gaseous NO is a major RNS, which acts as an oxidation-reduction signaling molecule with diverse physiological functions in plants. The half-life of NO in biological tissues is longer than that of ROS and is considered to be a relatively stable free radical [14]. NO was first proposed to participate in growth and diversion through its negative chemotaxis on lily (*Lilium longiflorum*) pollen tube growth [39]. Later, the pollen of several plants was discovered to produce NO [40]. In olive (*Olea europaea*), ROS and NO are produced in reproductive organs in a phased and tissue-specific manner. Stigma surface, anther tissue, pollen grains, and pollen tube are the tissues that accumulate the most ROS and NO [41]. During the receiving period, pollen grains and pollen tubes have an increased ability to produce NO. When NO is actively produced, the presence of ROS decreases [41]. These studies suggest that NO may evolve for intercellular communication tasks during the programming stage of sexual reproduction. There have been many studies on the role of RNS, especially NO, on the interaction of pollen and pistil, pollen tube growth, pollen tube rupture to release sperm, and fertilization [14,42,43]. For example, the arrival of Arabidopsis pollen tubes to the ovule triggers NO accumulation in filamentous organs. This process, which is FERONIA (FER)-dependent and mediated by de-esterified pectin, prevents multiple pollen tubes from penetrating the female gametophyte and averts polyspermia [43]. *S*-nitrosoglutathione (GSNO), the main biologically active form of RNS, is the major donor of NO. GSNO reductase (GSNOR) has been shown to metabolize GSNO and play a key role in NO signaling [22,44]. Knockout of the *GSNOR* gene in tomato increased the level of endogenous NO and S-nitrosylation, resulting in a decrease in total pollen, and a decrease in germination and vigor [45].

### 2.2. Role of Enzymatic Antioxidants in Pollen Development

In order to maintain the redox balance, plants have evolved a complete antioxidant mechanism. Many antioxidants are involved in the process of pollen development (Table 1), which can be divided into two categories: enzymatic antioxidants and non-enzymatic antioxidants [3]. Previous studies have shown that these plant antioxidants have precise temporal and spatial expression patterns and play crucial roles in the redox homeostasis and development of male reproductive tissues [15].

The main enzymatic antioxidants (Figure 1) include superoxide dismutase (SOD), peroxidase (POD), catalase (CAT), ascorbate peroxidase (APX), glutathione peroxidase (GPX), glutaredoxin (GRX), glutathione S-transferase (GST), thioredoxin (TRX), and peroxiredoxin (PRX) [3,22].

Among them, SOD, CAT, POD, and APX are considered to be classic and important antioxidant enzymes. The deletion of *CAT1/2/3* genes in Arabidopsis will not only cause severe vegetative growth defects, but also minor reproductive defects [46]. Maize *CAT1* is the only CAT expressed in mature pollen and the immature milky endosperm [47]. When compared to other tissues of the plant, pollen has a relatively low POD activity as well as low isoenzyme polymorphism [48]. However, POD may play a role in the interaction between pistil and pollen [48]. Many sterile line anthers show abnormal tapetal PCD and organelles, which may be due to abnormal transcription levels of antioxidant enzyme genes and excessive ROS that disrupt the balance of the antioxidant system [25,49]. In the early development of wheat sterile line pollen, the expression levels of *SOD, CAT, POD, APX,* and *GPX* are significantly up-regulated, and the enzyme activity increases rapidly [25].

GRXs and TRXs have been described as the main protein families responsible for the redox status of protein cysteine (Cys) residues within the cells, and these Cys residues are usually maintained in their reduced state within the cells [15,50,51]. ROXY proteins are conserved plant-specific GRXs. In several species such as Arabidopsis, wheat, and rice, ROXY proteins have been shown to play critical roles in regulating cell division, cell size, and early anther differentiation [52,53,54]. Roxy proteins regulate anther development by activating the transcription of TGACG (TGA) motif-binding factors (*TGA9* and *TGA10*) through cysteine modification. The double mutants *tga9/tga10* and *roxy1/roxy2* have fairly similar defects in the development of male gametes in Arabidopsis [55]. GhTRXL3-2 can interact with FLOWERING LOCUS T (GhFT) in cotton (*Gossypium hirsutum*), and heterologous overexpression of *GhTRXL3-2* in Arabidopsis results in early flowering [56]. Cystathionine β-synthase (CBS) domain-containing proteins are ubiquitous redox regulators in plants. In the Arabidopsis anthers, CBSXs, TRXs, and PRXs proteins are involved in anther dehiscence and pollen release via the control of H_2_O_2_ [57,58,59]. CBSX1 regulates TRX and lignin polymerization in the anther endothecium [57]. In addition, CBSX2 directly modulates TRX in chloroplasts, which affects the level of H_2_O_2_, thereby, affecting the expression of genes related to secondary cell wall thickening in anther development [58]. Recent study has further confirmed that deficient lignin deposition-mediated anther indehiscence is caused by insufficient ROS accumulation in Arabidopsis *CBSX3* RNAi plants [59].

PRXs are involved in various aspects of plant development and environmental response, and are usually related to lignin modification [60]. In Arabidopsis, *PRX9* and *PRX40* encode expansin peroxidase and cross-link extensins to contribute to the integrity of tapetum and microspore cell wall during anther development [61].

### 2.3. Role of Non-Enzymatic Antioxidants in Pollen Development

Non-enzymatic antioxidants (Figure 1) refer to small molecular compounds with antioxidant effects, including ascorbate (ASA), glutathione (GSH), proline, α-tocopherol (Ve), carotenoids, flavonoids, and phenolic acids (PhAs) [3,22].

The AsA-GSH cycle includes two interdependent redox pairs: AsA/dehydroascorbate (DHA) and GSH/glutathione disulfide (GSSG). They regulate cellular redox status and plant resistance [50,62,63]. APX oxidizes AsA to DHA, and GSH is used as an electron donor by dehydroascorbate reductase (DHAR) to convert DHA to the reduced form of AsA. GSSG can be recycled into GSH by glutathione reductase (GR) and GSH can also interact with ROS and is oxidized to GSSG [50,63]. GSH has a high concentration in plant cells while GSSG are usually kept at very low levels and can be rapidly sequestered in the vacuole [3,64,65]. The metabolically active high-ROS pollen would require high levels of antioxidant activity, particularly high activities of enzymes such as GR in order to maintain high GSH/GSSG ratios [66]. GSH accumulation is required for the initiation of the floral meristem and has a strong association with flowering time [67,68]. Metabolic analysis of soybean sterile line pollen has found that the ROS scavenging system is defective, and GSSG is the most decreased among amino acid derivatives [63]. The flowers of glutathione-deficient *cad2-1* mutants, which contain a low GSH/GSSG ratio, cause a high degree of oxidation of anthers, pollen grains, and pollen tubes in Arabidopsis [66]. However, an excess of antioxidants also affects pollen development. The accumulation of AsA in tomato plants is related to damage to the structure of floral organs, especially the development of anthers and pollen, leading to male sterility [69].

Metabolic studies on some sterile lines and specific mutants have shown that the decrease in the levels of specific metabolites, such as flavonols, proline, and polyamines (PAs), is related to the decrease in pollen fertility [70,71,72,73]. Flavonols are catalyzed and synthesized by flavonol synthase (FLS). *FLS1* is highly expressed in Arabidopsis flowers, and also exhibits flavonol accumulation [74]. Studies have shown that many MYB family transcription factors are closely related to the production and function of flavonols, and play vital roles in the development of stamens. There are two *FLS* genes involved in the spatiotemporal biosynthesis of flavonols in *Freesia hybrida* flowers, which are controlled by differential phylogenetic MYB regulators [73]. The accumulation of excessive ROS in Arabidopsis stamens of *myb21* mutants leads to defects in stamen development, which can be partially rescued by treatment with ROS inhibitors or overexpression of *FLS1* [75]. The proline needed for pollen development and reproduction is mainly synthesized in developing microspores and mature pollen grains [72]. The interruption of proline synthesis in Arabidopsis can lead to abortion and sterility during gametophyte development [70,76]. Prolyl Aminopeptidase 1 (PAP1) can release N-terminal proline. Loss of PAP1 function in plants reduces pollen fertility and sensitivity to osmotic stress [77]. PAs mainly exists in free form in higher plants, including putrescine (Put), spermidine (Spd), and spermine (Spm) [78]. S-adenosylmethionine decarboxylase (SAMDC) is a key enzyme for the synthesis of PAs. High levels of soluble SAMDC are found in male fertile anthers from the late stage of microspores, and there are no soluble SAMDC in male sterile anthers [71]. PAs, especially Spd, maintain high levels throughout the pollen development of kiwifruit (*Actinidia deliciosa*), and decline in the final stage of sterile pollen development [71].

**Table 1:** Role of various antioxidants during flower development in different plant species.

Plant Species	Related Antioxidants	Functional Period	References
Arabidopsis	CAT	Pollen development	[46,47]
	TRX, GSH	Development of flower buds	[68,79,80]
	GSH, GSSG	Flower development and pollen vigor	[66]
	CC-type GRX	Petal and anther development	[52,53,54,55]
Tomato	TRX, PRX	Microspore development, anther secondary cell wall thickening and anther dehiscence	[57,58,59,61]
	Flavonols	Stamen development, pollen wall formation and late flower development	[73,75,81]
	Proline	Pollen development	[70,76,77]
	ASA	Development of anthers and pollen	[69]
Maize	CAT, SOD	Pollen development	[82]
Wheat	SOD, CAT, POD, APX, GPX	Pollen development	[25,49]
Soybean	CAT, POD; GSSG, Flavonols	Pollen development and germination	[63]
Kiwifruit	PAs	Pollen development and germination	[71]

## 3. The Protective Mechanism of Antioxidant in Pollen Development under Capricious Environment

**Figure 2:**
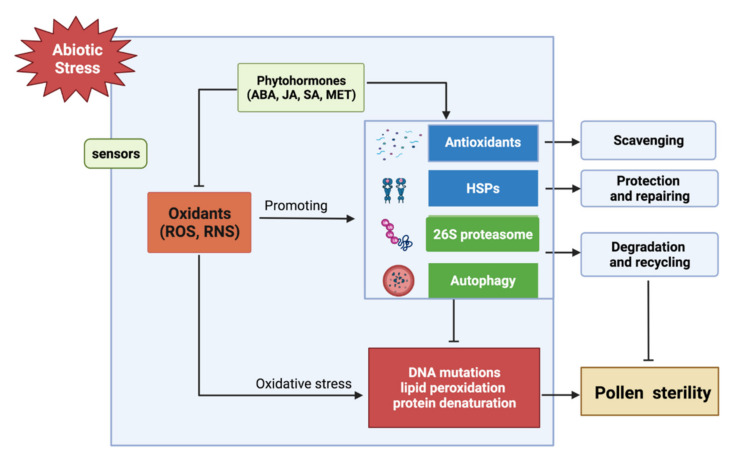
Regulation of redox balance in response to abiotic stresses. Abiotic stresses can induce reactive oxygen species (ROS) bursts, causing damage to cell structure (membrane lipids) and biomolecules (DNA, proteins), leading to pollen sterility. At the same time, different signal transduction and regulatory networks are induced, thereby improving the ability to scavenging oxidants, enhancing the protection and repair capabilities of Heat Shock Proteins (HSPs), and promoting the degradation of denatured proteins mediated by 26S proteasome and autophagy, ultimately achieving the goal of reducing the damage of oxidative stress to cells and improving the fertility of pollen.

Abiotic stresses such as high temperature, drought, and cold have negative impacts on the reproductive development of plants, and pollen is particularly sensitive to environmental stimuli during the entire development process. Oxidative stress induced by abiotic stress can trigger excessive accumulation of ROS and cause serious damage to cells, including protein denaturation, lipid peroxidation, and DNA mutations, even PCD [17]. It ultimately affects the quantity and morphology of pollen, cell wall structure, and importantly, pollen metabolism, leading to pollen abortion and male sterility during anther development [18,83]. Plants have integrated antioxidant protection systems in response to environmental stimuli (Figure 2). The antioxidant system, protein degradation and regeneration system, and phytohormones and peptides all participate in the adaptation of pollen to abiotic stress by mediating a wide range of adaptive responses [2,17,84].

### 3.1. Regulation of Redox Balance

The activity/content of antioxidants and specific metabolites increases to detoxify ROS and protect anther development under different abiotic stresses (Table 2). Among all antioxidant enzymes, CAT exhibits one of the highest turnover rates to catalyze H_2_O_2_ to H_2_O in an energy-efficient manner [85]. Therefore, CAT plays an indispensable role in the detoxification of excess ROS produced under abiotic stresses [86]. During meiosis, the APX and GR activities in rice anthers are relatively less sensitive to high temperature than the CAT and SOD activities in anthers [87]. Under drought stress, the antioxidant enzyme activities of drought-resistant wheat varieties, including SOD, POD, APX, GR, and CAT, were higher than those of drought-sensitive varieties, thereby removing excess ROS and protecting anther development [88]. Non-enzymatic antioxidant molecules also participate in the oxidative protection of pollen. PAs is a regulator of redox homeostasis, which can increase the activities of antioxidant enzymes and is also one of the sources of ROS [78]. Flavonols are considered to be powerful antioxidants for the detoxification of ROS [89,90]. The accumulation of pollen-specific flavonols promotes drought-induced male fertility by eliminating ROS in Arabidopsis [75]. The accumulation of flavonols in tomato plants under heat stress is much higher than that under the combination of salinity and heat [91,92]. Under cold stress, treatment with Spd can regulate Ca^2+^, ROS homeostasis, and cell wall deposition to reorganize the growth pattern of pollen tubes and significantly increase pollen germination rate and pollen tube length [93].

In addition to changes in metabolic activity and substances, antioxidant-related transcription levels and protein levels are also induced by stresses. *Cu/Zn-SODa* is sensitive to heat stress and is closely related to the down-regulation of *cCu/Zn-SOD1* expression, thus having a great influence on the total SOD activity. [94]. High temperature exposure during meiosis significantly affects the expression and activity of SOD, CAT, and POD in developing anthers, but has no significant effect on APX and GR [87]. Under heat stress, the decrease in CAT activity in rice anthers is mainly due to the rapid inhibition of *OsCATB* transcripts, which is one of the factors leading to the increase in ROS accumulation in anthers and the decrease in pollen vigor [87]. Transcription factor *MYB Important for Drought Response 1* (*MID1*) is expressed in rice anthers and is further induced by drought stress. In *MID1* overexpression plants, the expression of *POD* and *SOD* is increased, which causes an increase in ROS scavenging capacity to protect the tapetum cells and enhance drought tolerance [95]. Cold stress causes ROS accumulation, which leads to tapetum degradation and pollen abortion. A point mutation of the *Low Temperature Tolerance 1* (*LTT1*) gene prevents ROS overproduction, allowing for correct pollen development, thereby improving the cold tolerance and seed setting ability of rice [96].

As important signal molecules, phytohormones also participate in the regulation of redox state in anther and pollen under abiotic stresses (Figure 2). Heat stress at the meiotic stage of pollen disturbs ROS and sugar homeostasis, leading to pollen sterility in rice, which can be reversed by abscisic acid (ABA) [97]. Rice *Drought-Induced LTP* (*OsDIL*) is mainly expressed in anthers and responds to drought and ABA. OsDIL regulates the drought resistance of rice during reproductive development by up-regulating the expression of *SOD*, *POD,* and ABA synthesis gene *Zeaxanthin Epoxidase 1 (ZEP1)* [98]. Auxin reduces the membrane lipid peroxidation and ROS accumulation, as well as affecting the underlying antioxidant enzyme activities in rice spikelets under drought stress [99]. Jasmonic acid (JA) actively regulates ROS production, the antioxidant system, and amylase activity in the spikelets of sterile rice to alleviate the spikelet opening obstacles during the flowering period under heat stress [100]. Salicylic acid (SA) is known as a phenolic compound and hormone-like substance. Under heat stress, SA can enhance the antioxidant ability of rice plants to maintain the redox state by scavenging excess ROS, and prevent the degradation of the tapetum [101].

**Table 2:** Regulatory function of antioxidants in pollen development under different stresses.

Stress	Plant Species	Related Antioxidants	Stress Damage	References
Heat	Rice	CAT, SOD, POD	Decline in pollen viability and spikelet fertility	[87,94]
	Rice	ASA	Spikelet-opening impairment	[100]
	Tomato	GST, APX, Flavonoids, PAs	Microspore abortion and low pollen germination rate	[92,102,103]
Drought	Wheat	SOD, POD, APX, CAT	Male fertility and grain number reduction	[88]
	Rice	SOD, POD	Tapetal defects and reduced pollen fertility	[95,98]
	Arabidopsis	Flavonols	Stamen defects and reduced fertility	[75]
	Rice	SOD, POD	Severe tapetal defects and reduced pollen fertility	[96]
	Camellia	Spd	Pollen germination rate and pollen tube length	[93]
Cold	Chickpea	Proline, GPX, GSH	Disruption of gamete development and pollen sterility	[104]

### 3.2. Maintenance of Protein Homeostasis

Proteomics studies have shown that mature pollen pre-synthesizes a large number of proteins with predefined functions, such as cell wall metabolism, energy metabolism, signal transduction, etc. [105]. Oxidative stress caused by abiotic stresses usually changes protein stability, membrane fluidity, the cytoskeleton, and metabolic balance in anther and pollen development. Therefore, maintaining the stability of functional proteins is very important for pollen development.

HSPs function by re-stabilizing protein conformation under stress conditions (Figure 2). HSPs and ROS detoxification systems participate in the crosstalk between different stress signaling transduction networks to enhance plant resistance under abiotic stresses [106,107]. After short-term heat stress in many species, different types of HSPs, such as small HSPs, HSP70, HSP90, and HSP100, are accumulated in the developing anthers and pollen grains [105,108]. Melatonin (MET), a novel signaling molecule in plant response to stresses, promotes the high levels of *HSPs* and *Autophagy-Related Genes* (*ATGs*) transcripts and induces autophagic signaling in tomato anthers, and ultimately increases pollen viability and germination under heat stress [109].

Oxidative stress results in massive protein denaturation and cytoplasmic organelle damage. The removal of denatured proteins and organelles is essential for cell survival [110]. The ubiquitin–proteasome system and autophagy (Figure 2) are the two main proteolytic systems involved in degradation of misfolded and damaged proteins and organelles [111,112]. Loss of 26S proteasome function leads to a series of plant development defects. For example, loss function of RPT2 subunit, a component of the 26S proteasome, impairs plant growth and reproductive development in Arabidopsis [113]. Studies have reported that many genes encoding ubiquitin–conjugating enzyme (E2) and ubiquitin ligase (E3) are stress-induced and can regulate the tolerance of plant reproductive organs to a variety of abiotic stresses [114,115,116]. The RING-type E3 ligase High Expression of Osmotically Responsive Genes1 (HOS1) can ubiquitinate flowering-promoting transcription factor CONSTANS (CO), which is then degraded by the proteasome [117,118]. Exposure to low temperature promotes the degradation of CO by the HOS1-dependent proteasome and leads to FT inhibition, thereby delaying flowering [117,118]. Autophagy is also required in the tapetal PCD during the development and maturation of pollen, and participates in plant responses to abiotic stresses [119,120]. Moreover, autophagy is also involved in the breakdown of liposomes and the transfer of lipids from tapetum cells to the surface of microspores in rice [121]. Autophagy-deficient mutants in Arabidopsis and rice are reported to be hypersensitive to oxidative stress. Therefore, autophagy, which is induced during tapetal PCD and pollen development, plays critical roles in organelle quality control and environmental stress response [121,122]. In short, the protein quality control mediated by 26S proteasome and autophagy plays a decisive role in pollen development and stress resistance. There are many overlaps between these two protein regulatory mechanisms, and their different roles in specific conditions need to be further studied.

## 4. Potential Agricultural Application of Antioxidants for Normal Pollen Development under Stresses

**Figure 3:**
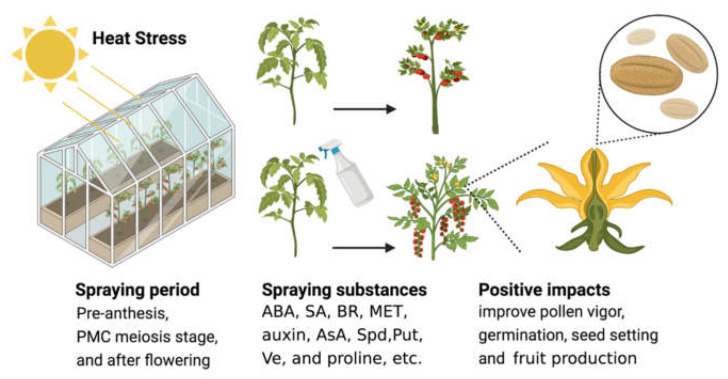
Regulation of redox balance in response to different abiotic stresses. In many plants (tomato, rice, wheat, and soybean), exogenous spraying of many antioxidant regulators at different stages of flower development can effectively enhance the resistance of plants and pollen under abiotic stresses, improve pollen vigor, germination rate, fruit setting rate, and yield.

In agricultural production, exogenous spraying of plant growth regulators (PGRs) such as auxin, ABA, SA, BR, and MET can increase plant resistance under biotic and abiotic stresses, reduce damage to the tapetum layer, and improve pollen vigor and fertility (Figure 3) [97,109,123,124,125,126,127,128]. In addition to PGRs, there are other antioxidant sources such as proline, Put, Spd, Ve, and AsA that also play important roles in plant growth, development, and stress resistance [43,72,129,130,131,132,133,134,135,136,137].

Exogenous application of auxin improves the tolerance of developing pollen to continuous mild heat stress in Arabidopsis and barley (*Hordeum Vulgare* L.) [123]. ABA spraying on the leaf surface during the meiotic phase of pollen mother cell significantly reduces the pollen sterility caused by heat stress in rice [97]. Pretreatment with SA could alleviate damage of the spikelet differentiation during the floret differentiation stage of rice under heat stress [124]. The application of ethylene releaser before the short-term heat stress treatment improves tomato pollen thermotolerance, whereas application of an ethylene inhibitor reduced this [125]. Exogenous application of 24-epibrassinolide (EBR) and ecdysone analogs (7,8-dihydro-8α-20-hydroxyecdysone; DHECD) can effectively improve pollen vigor and germination, and reduce pollen bursting in rice [126]. BRs also promote spikelet development through increasing antioxidant system levels and energy charge during panicle development in rice [127]. Recent studies have found that exogenous MET alleviates ROS production and induces activity of antioxidant enzymes under heat stress in tomato [109]. The application of exogenous MET significantly improves the translocation of carbon assimilates to drought-stressed anthers in cotton, which helps to produce more ATP for reproductive activities, finally improving male fertility [128].

Proline is extensively involved in the response to various abiotic stresses [129,130]. Supplementing proline to mung bean (*Vigna radiata*) plants significantly increases its endogenous concentration in leaves, anthers, pollen grains, and ovules, and significantly improves reproductive functions under heat stress [131]. Studies have also shown that excessive production of proline in Arabidopsis pollen may increase the fertility of pollen and increase crop yields under unfavorable conditions [70,72]. Therefore, inhibiting the biosynthesis of proline in pollen can be used to create male sterility [70,72]. Exogenous application of Spd apparently alleviates heat stress-induced tapetum damage in rice [132,133,134]. In addition, exogenous Spd and Put increase their endogenous levels and improve antioxidant capacity and membrane stability, the spikelet fertility, seed setting rate, and ear yield in rice under heat stress [43,134,135]. Pre-anthesis ASA treatment before heat stress enhances the thermotolerance capacity of wheat pollens [137]. Exogenous application of Ve, glycine betaine, and SA also increases the antioxidant levels, prevents oxidative damage to plant membranes, and significantly improves the pollen germination and spikelet fertility of rice [136]. Anyway, PGRs and substances can play a regulatory role in a more refined and efficient manner; thereby, increasing plant resistance and yield; however, only a few have been promoted in actual production. Therefore, it is very necessary to develop more broad-spectrum and practical formulas and to increase the scale of practical applications in production.

## 5. Conclusions and Prospective

In the process of growth and development, plants are constantly stimulated by various environmental cues, which interfere with their redox homeostasis. In order to perform normal developmental activities, plants have evolved complex and sophisticated redox regulation systems. These redox processes are continuously happening inside the plant cells within certain variable range. Any deviation in redox balance within the adjustable range can trigger plant signaling cascades through various biomolecules. These redox signals (ROS/RNS) regulate diverse aspects of male gametophyte development through their crosstalk with phytohormones and other signal transduction pathways. When subjected to abiotic stresses such as high temperature, the balance between oxidants and antioxidants becomes disturbed and this results in oxidative stress. Depending on the dosage of oxidants, the cellular structure and metabolism will be damaged to varying degrees, and eventually this will lead to microspore abortion and pollen sterility. By studying the redox system during stamen development under stressful conditions, the importance of their action/interaction may be clarified. This further improves our understanding on vital prospects of the redox signaling cascade in plant growth and development. In this review, we have gathered the recent information related to redox signaling in pollen development and stress response; however, there are still many questions/areas needing to be explored thoroughly. For example, (i) how the pollen development process and tapetal PCD are precisely regulated by the redox system; (ii) what other genes or biomolecules are involved, and how the crosstalk between different signal networks is unified in the process of pollen development; and (iii) are there any other special mechanisms for the regulation of pollen resistance under abiotic stresses. Moreover, many studies have shown that PGRs and antioxidants can help improve the plants’ resistance towards stresses and regulate flowers and fruit-setting rate. However, due to the complexity of actual production conditions, the abundance of crops, and the long period of field trials and extensions, their application in agricultural production is relatively limited. Clarifying these mechanisms can help us in developing more efficient, targeted, or universally adaptable regulatory substances to support sustainable agricultural production.

## Abbreviations

24-epibrassinolide	EBR
7,8-dihydro-8α-20-hydroxyecdysone	DHECD
abscisic acid	ABA
Anther Dehiscence Gene	ADR
ARGONAUTE 2	AGO2
ascorbate	AsA
ascorbate peroxidase	APX
Autophagy-Related Genes	ATGs
Brassinazole Resistant 1	BZR1
catalase	CAT
CONSTANS	CO
cytosolic protein response	CPR
cysteine Cys Cystathionine β-synthase	CBS
Defective tapetum cell death 1	DTC1
dehydroascorbate	DHA
dehydroascorbate reductase	DHAR
Drought-Induced LTP	DIL
endoplasmic reticulum-unfolded protein response	ER-UPR
Fatty Acid Export 1	FAX1
fatty acids	FAs
FERONIA	FER
Flavonol Synthase	FLS
FLOWERING LOCUS T	FT
glutathione	GSH
glutathione disulfide	GSSG
glutathione peroxidase	GPX
glutathione reductase	GR
glutathione S-transferase	GST
GSNO reductase	GSNOR
Heat Shock Protein	HSP
High Expression of Osmotically Responsive Genes1	HOS1
Hexokinase 1	HXK1
hydrogen peroxide	H_2_O_2_
hydroxyl radical	HO^•^
superoxide	O2^•−^
Inflorescence Deficient in Abscission	IDA
jasmonic acid	JA
lipid alkoxy	RO^•^
lipid peroxides	LOOH
Low Temperature Tolerance 1	LTT1
melatonin	MET
metallothioneins	MTs
male sterility 33	MS33
MYB Important for Drought Response1	MID1
nitric oxide	NO
nitrous oxide	N_2_O
peroxidase	POD
peroxiredoxin	PRX
peroxynitrite	ONOO^−^
phenolic acids	PhAs
plant growth regulators	PGRs
polyamines	PAs
programmed cell death	PCD
Prolyl Aminopeptidase 1	PAP1
putrescine	Put
reactive carbonyl species	RCS
reactive nitrogen species	RNS
reactive oxygen species	ROS
Respiratory Burst Oxidase Homologs	RBOHs
S-adenosylmethionine decarboxylase	SAMDC
salicylic acid	SA
S-nitrosoglutathione	GSNO
GSNO reductase	GSNOR
spermidine	Spd
spermine	Spm
superoxide dismutase	SOD
TGACG	TGA
thioredoxin	TRX
α-tocopherol	Ve
ubiquitin-conjugating enzyme	E2
ubiquitin ligase	E3
Zeaxanthin epoxidase 1	ZEP1
